# A Case of Suspected Isolated Follicle-Stimulating Hormone (FSH) Deficiency Where Spermatogenesis Was Acquired by Human Menopausal Gonadotropin (hMG)

**DOI:** 10.7759/cureus.36182

**Published:** 2023-03-15

**Authors:** Miho Sugie, Hatsuki Hibi

**Affiliations:** 1 Urology, Kyoritsu General Hospital, Nagoya, JPN

**Keywords:** hmg, male infertility, isolated follicle-stimulating hormone (fsh) deficiency, fsh, azoospermia

## Abstract

Isolated follicle-stimulating hormone (FSH) deficiency is a rare cause of infertility in both sexes, and only a few cases have been reported in Japan. This is a case report of a young male patient with isolated FSH deficiency and azoospermia who was successfully treated with human menopausal gonadotropin (hMG).

A 28-year-old male patient was referred for azoospermia. The delivery at his birth was uneventful and a family history of infertility or hypogonadism was not observed. The testes volume was 22/24 mL (right/left). No varicocele was observed in the ultrasound, and no sign or symptom of hypogonadism was found. In the semen analysis, however, the sperm concentration was as low as 2.5×10^6^/mL and the motility was less than 1%. The endocrine panel revealed luteinizing hormone (LH) (2.1 mUI/mL, normal values 0.8-5.7 mUI/mL) and testosterone (6.57 ng/ml, normal values 1.42-9.23 ng/mL) were normal, while the FSH level was very low (0.6 mUI/mL, normal values 2.0-8.3 mIU/mL). The odor and the karyotype 46, XY, were normal. The brain MRI scans showed no abnormal findings. Genitalia and potency were normal. The diagnosis was made of isolated FSH with severe oligoastenozoospermia clinically.

FSH replacement therapy was employed. The patient self-injected 150 units of hMG three times a week. After 3 months of the treatment, the sperm concentration and motility went up to 264×10^6^/mL and 12%, respectively. At 5 months, the patient’s spouse conceived naturally, and at 7 months the treatment was terminated. During the treatment, FSH rose to the normal range, while other test items showed no change. The patient’s health condition was uneventful. The spouse delivered a healthy boy.

In conclusion, for isolated FSH with severe oligoastenozoospermia, hMG can be as effective as recombinant human FSH (rh-FSH), although the dosage remains a matter of discussion.

## Introduction

Follicle-stimulating hormone (FSH), released by the anterior pituitary gland, is an important hormone involving reproductive functions. It is a major stimulator of seminiferous tubule growth, binding to Sertoli cells and spermatogonial membranes in the testis. Isolated FSH deficiency was initially described in the 1970s, and the first case was reported of a 22-year-old woman with primary amenorrhea in Israel [1,2] The disease can be found in infertile men, especially in azoospermic patients. Here we report a clinical case of a young male infertile patient with suspected isolated FSH deficiency and azoospermia, who was successfully treated with human menopausal gonadotropin (hMG).

## Case presentation

A 28-year-old male patient was referred to our male infertility unit for azoospermia. At his birth, the delivery was uneventful and a family history of infertility or hypogonadism was not observed. He was 178 cm tall and weighed 78 kg, and his body mass index (BMI) was 24.5 kg/m2.

On physical examination, the testes' volume was 22/24 mL (right/left) and the testes were normal in texture. No varicocele was observed. The tanner stage of pubic hair and penis was normal in virilization, with no sign or symptom of hypogonadism.

In laboratory investigations, semen analysis revealed that the sperm concentration and motility were 2.5×10^6^/mL and less than 1%, respectively. All blood tests were normal. The endocrine panel revealed LH (2.1 mUI/mL, normal values 0.8-5.7 mUI/mL) and testosterone (6.57 ng/ml, normal values 1.42-9.23 ng/mL) were normal, while the FSH level was very low (0.6 mUI/mL, normal values 2.0-8.3 mIU/mL). In the diagnostic workup, the odor was normal and so was the karyotype 46, XY. No abnormal findings were observed in the brain MRI scans. Genitalia and potency were normal.

A diagnosis was made of isolated FSH deficiency with severe oligoastenozoospermia clinically, and FSH replacement therapy was employed. The patient self-injected 150 units of hMG three times a week. In his case, recombinant human (rh-FSH) was not used due to its high cost. After 3 months of treatment, the sperm concentration and motility went up to 264×10^6^/mL and 12%, respectively; blood tests revealed normal erythrocyte and liver functions. The changes in endocrine panel are shown in Table 1.

**Table 1:** Endocrine panel LH: luteinizing hormone; FSH: follicle-stimulating hormone

	Before	At 3 months
LH (mUI/mL)	2.1	1.7
FSH (mUI/mL)	0.6	7.1
Total testosterone (ng/mL)	6.576	6.173
Free testosterone (pg/mL)	16.8	12.3

At 5 months of treatment, the patient’s spouse conceived naturally, and the treatment was terminated at 7 months. At 3 months after the termination, the sperm concentration remained normal, though the sperm motility remained low. (Table2).

**Table 2:** Semen analysis showing sperm concentration and motility

	Before	1 month	3 months	4 months	3 months after termination
Concentration (/mL)	2.5×10^6^	14×10^6^	264×10^6^	264×10^6^	102×10^6^
Motility (％)	1	6	12	7	11

During the treatment, the patient’s health condition was uneventful. The spouse delivered a healthy boy.

## Discussion

Isolated FSH deficiency is a rare cause of infertility in both sexes, and only a few cases have been reported in Japan. This is a case report of a young male patient with isolated FSH deficiency and azoospermia, who was successfully treated with human menopausal gonadotropin (hMG). FSH is essential to initiate spermatogenesis at puberty, and its major physiologic role is to stimulate spermatogenesis to a quantitatively normal level in adults. FSH also stimulates the production of inhibin, a protein hormone produced by Sertoli cells that provides negative feedback to the pituitary and hypothalamus [3].

Isolated FSH deficiency is asymptomatic except for low sperm quality and, in this sense, very different from male hypogonadotropic hypogonadism (MHH), such as decreased libido, osteoporosis, gynecomastia, and eunuchoidism [4]. In most cases of men, therefore, it is found together with azoospermia or oligoasthenozoospermia, as is the case in ours.

Usually, luteinizing hormone releasing hormone (LH-RH) test and gonadotropin-releasing hormone (GnRH) test are required for a definitive diagnosis of isolated FSH deficiency. For financial reasons, however, the patient in this report refused to undergo either of the tests. Furthermore, he refused to measure Inhibin B also for financial reasons. Therefore, a definitive diagnosis of isolated FSH deficiency was not made in this case.

The main treatment of Isolated FSH deficiency is by FSH replacement, and the choice of drug is recombinant human FSH (rh-FSH) or hMG. Whereas rh-FSH is pure FSH and does not have systemic effects as LH and testosterone do, hMG contains a small amount of LH and may cause adverse effects including elevated testosterone, polycythemia, liver function disorder, and acne. In terms of cost, however, rh-FSH is very expensive and hMG is practically preferred [5]. In our case too, the patient chose hMG for an economic reason. Fortunately, the patient showed no adverse effects.

Through FSH replacement, spermatogenesis can be gained in most cases. A case was reported where a natural pregnancy occurred after 5 months of treatment of rh-FSH [1]. In the present case, in 3 months of hMG treatment, the patient’s spermatogenesis and appearance of motile sperm in the ejaculates improved and the patient’s spouse conceived naturally at 5 months of his treatment. This result indicates that hMG can be as effective as rh-FSH.

## Conclusions

In the present case, the treatment was terminated shortly after the spouse conceived. Supposedly, the patient's spermatogenesis would decrease slowly due to the lack of FSH, but when that really happened is unknown because the patient was not followed up. The dose, duration, and timing of termination are a matter of future discussion. A clinical implication is that, although the cause of isolated FSH remains unclear, regular check-ups for semen analysis would help in diagnosing the condition.
